# Seroprevalence and associated factors of *Helicobacter pylori* among Somali University students: a cross-sectional study

**DOI:** 10.3389/fpubh.2026.1753892

**Published:** 2026-03-18

**Authors:** Abdulrazak Mohamed Ahmed Qudde, Abdisamad Sh. Yusuf Mohamod, Abdullahi Mohamed Hassan Fujeyra

**Affiliations:** Faculty of Medicine and Health Sciences, Somaville University, Mogadishu, Somalia

**Keywords:** associated factors, ELISA, *Helicobacter pylori*, seroprevalence, Somalia, university students

## Abstract

**Background:**

*Helicobacter pylori (H. pylori)* seropositivity remains a major public health concern in low and middle-income countries, including Somalia. Young adults, particularly university students, represent an understudied group despite potential exposure to lifestyle and environment-related risk factors. This study assessed the seroprevalence of *Helicobacter pylori* IgG antibodies and associated factors among students at Abrar University in Mogadishu, Somalia.

**Methods:**

A cross-sectional study was conducted among 149 students. Serum IgG antibodies were tested using ELISA which reflects previous exposure rather than active infection. Data on demographics, dietary habits, sleep patterns, and environmental exposures were collected using a validated questionnaire. Univariable logistic regression was used to assess associations.

**Results:**

The overall *H. pylori* seroprevalence was 56.4%. Associations were observed between seropositivity and spicy food consumption (OR = 2.1, *p* = 0.05), irregular sleep (OR = 1.8, *p* = 0.02), and shared drinking water sources (OR = 1.7, *p* = 0.04).

**Conclusion:**

*H. pylori* seropositivity was common among the study population. Lifestyle and environmental factors were associated with seropositivity; however, results should be interpreted cautiously as serology does not indicate active infection.

## Introduction

*Helicobacter pylori* (*H. pylori*) is a Gram-negative, microaerophilic bacterium capable of colonizing the human gastric mucosa. Since its discovery by Warren and Marshall in 1983, *H. pylori* has been recognized as one of the most common chronic bacterial infections worldwide and a major contributor to gastrointestinal diseases, including chronic gastritis, peptic ulcer disease, and gastric malignancies. Although many infected individuals remain asymptomatic, *H. pylori* is a well-established risk factor for gastric cancer and mucosa-associated lymphoid tissue (MALT) lymphoma, making early detection and prevention a public health priority (1).

Globally, approximately 4.4 billion individuals are estimated to be infected with *H. pylori*, with the highest prevalence reported in low and middle-income countries (LMICs). Africa reports particularly high prevalence rates, ranging from 50% to over 80% in some regions. High prevalence in LMICs is often attributed to overcrowding, limited sanitation infrastructure, unsafe water sources, and low socioeconomic conditions. Transmission typically occurs during childhood and can persist throughout life in the absence of appropriate treatment. High prevalence in African settings has been documented for decades (1, 2).

In Somalia, few studies have addressed the prevalence of *H. pylori*, and existing research has primarily focused on children and symptomatic adults, revealing high infection rates consistent with regional patterns. However, young adults—particularly university students—remain largely understudied. This demographic is of particular interest because lifestyle and environmental exposures, such as communal living conditions, shared water sources, irregular dietary habits, and disrupted sleep patterns, may increase susceptibility to *H. pylori* infection. Understanding infection patterns in this group can inform public health interventions targeted at young adults, potentially reducing transmission within households and communities.

Previous studies in neighboring East African countries have reported high *H. pylori* prevalence among young adults. Studies among Ethiopian university students similarly reported high prevalence (3). Comparable screening among university students in West and East Africa showed similar patterns (4, 5). For instance, prevalence rates of 62% in Ethiopia, 58% in Kenya, and 68% in Sudan highlight the vulnerability of university-age populations to infection. Lifestyle factors such as consumption of spicy foods, irregular sleep patterns, and reliance on shared water sources have been implicated as potential risk factors, although evidence remains limited and sometimes contradictory (6–9). Moreover, most studies rely on serological tests that detect IgG antibodies, which may reflect past exposure rather than current infection. Nevertheless, serological testing remains useful for estimating the burden of *H. pylori* in population-based studies where more invasive diagnostic methods are not feasible (10).

Despite the recognized importance of *H. pylori* infection in gastrointestinal health, there is a paucity of data among Somali university students. This knowledge gap limits the development of targeted interventions to reduce infection risk and prevent long-term complications such as gastritis, peptic ulcer disease, and gastric cancer. Addressing this gap can also provide baseline data for future longitudinal and interventional studies.

### Study aim

This study aimed to determine the prevalence of *H. pylori* seropositivity among students at Abrar University in Mogadishu, Somalia, and to identify associated lifestyle and environmental risk factors, including dietary habits, sleep patterns, and sources of drinking water. By focusing on this understudied population, the study seeks to provide evidence-based recommendations for public health interventions to reduce the burden of *H. pylori* among Somali young adults.

## Methods

### Study design and setting

A cross-sectional study was conducted at Abrar University, Mogadishu, Somalia, from January to March 2025. The study targeted undergraduate students across five faculties: Health Sciences, Engineering, Economics, Agriculture, and Veterinary Medicine. The primary objective was to estimate the prevalence of *H. pylori* seropositivity and identify associated risk factors, including dietary habits, sleep patterns, and water source exposure.

### Sample size calculation

The sample size was calculated using the single population proportion formula: *n* = Z^2^ × P × (1 − P)/*d*^2^. Where: *n* = required sample size. Z = Z-score for 95% confidence interval (1.96). *P* = estimated prevalence of *H. pylori* (50% assumed due to lack of prior data). *d* = margin of error (0.05 for 5%). *n* = (1.96) ^2^ × 0.5 × (1–0.5)/(0.05) ^2^ = 385. Since the total population was finite (*N* = 243), the sample size was adjusted using the finite population correction: *n* = n₀/[1 + (*n*₀ − 1)/N] = 149. Therefore, the final required sample size after applying the finite population correction was 149 participants. Thus, a total of 149 students were included in the study.

### Sampling procedure

A simple random sampling technique was employed. A complete list of eligible undergraduate students aged 18–30 years was obtained from the university registry. The total student population during the study period was 243. Using proportional allocation across faculties, participants were selected through computer-generated random numbers. Selected students were contacted and invited to participate. If a selected student declined, another student was randomly selected from the remaining list.

### Inclusion and exclusion criteria

*Inclusion criteria*: All undergraduate students aged (18–30 years), willing to participate and provide informed consent.

*Exclusion criteria*: Students with a history of *H. pylori* eradication therapy within the last 6 months or those with incomplete questionnaire responses were excluded.

### Data collection

Data were collected using a structured, self-administered questionnaire. The questionnaire was developed for the study and pretested on 20 students to ensure clarity and relevance. It collected information on:

Demographic characteristics (age, sex, faculty). Dietary habits (frequency of meals, consumption of spicy foods). Lifestyle factors (sleep patterns, smoking, physical activity) Environmental exposures (shared drinking water, household sanitation).

Validation: Content validity was ensured through expert review, and internal consistency was checked using Cronbach’s alpha (*α* = 0.78).

### Definition of key variables

Irregular sleep: Sleeping at inconsistent times (late sleeping) on most nights. Spicy food consumption: Self-reported regular consumption of spicy meals ≥3 times per week. Shared drinking water: Use of communal water sources (wells, taps, or fountains shared with multiple households).

### Sample collection and laboratory analysis

Venous blood samples (5 mL) were collected from each participant using aseptic techniques. Serum was separated by centrifugation at 3000 rpm for 10 min and stored at −20 °C until analysis.

### ELISA testing

The presence of *H. pylori* IgG antibodies was determined using a commercially available ELISA kit (Manufacturer: Bio Kit, Barcelona, Spain; Catalog #: BK-HpIgG-2022). The assay was performed according to the manufacturer’s instructions. Optical density was measured at 450 nm using a microplate reader. A cut-off value of ≥1.1 was considered positive, ≤0.9 negative, and 0.9–1.1 equivocal (samples in the equivocal range were retested).

According to the manufacturer, the assay sensitivity and specificity were reported as 94.5% and 92.8%, respectively. Positive and negative controls were included in each assay batch to ensure quality control.

### Data analysis

Data were entered into SPSS version 22 (IBM Corp., Armonk, NY, United States) and checked for completeness and consistency prior to analysis. Categorical variables were coded as binary indicators (e.g., gender: 0 = female, 1 = male; sleep pattern: 0 = regular, 1 = irregular; spicy food consumption: 0 = no, 1 = yes; shared drinking water source: 0 = no, 1 = yes).

Descriptive statistics were computed and summarized as frequencies and percentages. Univariable logistic regression analysis was performed to assess associations between independent variables and *H. pylori* seropositivity. Crude odds ratios (ORs) with 95% confidence intervals (CIs) were calculated.

Multivariable regression was not performed due to the exploratory nature of the study and considerations regarding model stability given the sample size. Therefore, the findings should be interpreted cautiously, and residual confounding cannot be excluded.

Statistical significance was set at *p* < 0.05 (two-tailed).

### Ethical considerations

This study was approved by the Ethics Review Committee of Abrar University, Mogadishu, Somalia (Approval No. AUEC10521, approved December 2024). Written informed consent was obtained from all participants prior to data collection. All procedures were conducted in accordance with institutional ethical guidelines and the Declaration of Helsinki. Participant confidentiality was maintained, and all data and biological samples were anonymized before analysis.

## Results

### Participant characteristics

Out of 150 students recruited, 149 completed all study procedures (one participant did not respond to the questionnaire). The mean age was 21.8 ± 2.1 years. The majority of participants were male (*n* = 75, 50.3%) and enrolled in the Health Sciences faculty (*n* = 48, 32.2%). Table 1 summarizes the demographic and lifestyle characteristics of the study participants.

**Table 1:** Demographic and lifestyle characteristics of participants (*N* = 149).

Variable	Category	Frequency (*n*)	Percentage (%)
Gender	Male	75	50.3
	Female	74	49.7
Age (years)	18–21	61	40.9
	22–25	72	48.3
	26–30	16	10.7
Faculty	Health Sciences	48	32.2
	Engineering	34	22.8
	Economics	27	18.1
	Agriculture	23	15.4
	Veterinary	17	11.4
Sleeping pattern	Early sleepers	69	46.3
	Late sleepers	80	53.7
Eating frequency	Once/day	22	14.8
	Twice/day	78	52.3
	Three times/day	49	32.9
Spicy food consumption	Yes	114	76.5
	No	35	23.5
Shared drinking water source	Yes	94	63.1
	No	55	36.9

### Prevalence of *H. pylori* Seropositivity

The overall seroprevalence of *H. pylori* seropositivity was 56.4% (*n* = 84). There was no statistically significant difference between males (48/75, 64.0%) and females (36/74, 48.6%) (*p* = 0.07). Table 2 shows the association between *H. pylori* seropositivity and participant characteristics.

**Table 2:** *H. pylori* seropositivity by gender and selected characteristics (*N* = 149).

Variable	Category	Positive *n* (%)	Negative *n* (%)	*p*-value
Gender	Male	48 (64.0)	27 (36.0)	0.07
	Female	36 (48.6)	38 (51.4)	
Sleeping pattern	Regular sleep patterns	35 (50.7)	34 (49.3)	0.02
	Irregular sleep patterns	49 (61.3)	31 (38.7)	
Spicy food consumption	Yes	74 (64.9)	40 (35.1)	0.05
	No	10 (28.6)	25 (71.4)	
Shared drinking water source	Yes	58 (61.7)	36 (38.3)	0.04
	No	26 (47.3)	29 (52.7)	

### Factors associated with *H. pylori* Seropositivity

Binary logistic regression analysis was performed to identify factors associated with *H. pylori* seropositivity. Table 3 presents the odds ratios (OR) and 95% confidence intervals (CI) for each risk factor.

**Table 3:** Logistic regression analysis of factors associated with *H. pylori* seropositivity.

Associated Factor	OR	95% CI	*p*-value
Male gender	1.6	0.9–2.8	0.07
Late sleeping pattern	1.8	1.2–2.7	0.02
Spicy food consumption (Yes)	2.1	1.1–3.9	0.05
Shared drinking water source	1.7	1.0–2.9	0.04

*Statistically significant.

### Summary of key findings

- Overall seroprevalence of *H. pylori* was high (56.4%), consistent with regional data.
- Irregular sleeping patterns, spicy food consumption, and reliance on shared drinking water were significantly associated with higher odds of seropositivity.
- No significant gender difference was observed.
- Findings highlight modifiable lifestyle and environmental associated factors relevant for public health interventions among university students in Somalia.

## Discussion

This study assessed the seroprevalence of *Helicobacter pylori* IgG antibodies and factors associated with seropositivity among university students at Abrar University in Mogadishu, Somalia. The overall seroprevalence of 56.4% indicates widespread prior exposure to *H. pylori* within this young adult population and is consistent with findings reported from other East African and urban Somali settings. These results suggest that exposure to *H. pylori* remains common among young adults in low-resource environments (3, 5).

### Prevalence and gender distribution

Although a higher proportion of male students were seropositive compared with females, this difference was not statistically significant. This finding aligns with previous studies indicating that gender alone is not consistently associated with *H. pylori* seropositivity among young adults. Variability across studies may reflect differences in environmental exposures, living conditions, or behavioral patterns rather than biological susceptibility (3, 4).

### Lifestyle and environmental associations

Irregular sleep patterns were associated with higher odds of *H. pylori* seropositivity. This association should be interpreted cautiously. Given the cross-sectional design, temporal direction cannot be established, and reverse causation cannot be excluded. Irregular sleep may also co-occur with other unmeasured lifestyle or socioeconomic factors that influence exposure risk (6, 8).

Frequent consumption of spicy foods was also associated with seropositivity. This finding does not imply that spicy food intake causes *H. pylori* infection. Rather, spicy food consumption may reflect broader dietary practices or cultural habits that correlate with exposure. Similar associations have been reported in studies from the Middle East and parts of Africa, although evidence remains inconsistent across settings (6, 9).

Use of shared drinking water sources was associated with increased odds of seropositivity, supporting existing evidence that communal or potentially unsafe water sources may be linked to *H. pylori* exposure in low-resource environments. However, water quality was not directly assessed, and residual confounding related to sanitation or household conditions cannot be ruled out (2, 5).

### Interpretation in the context of serological testing

An important consideration is that ELISA-based IgG testing reflects previous exposure rather than active infection. Consequently, the associations observed in this study relate to cumulative exposure over time rather than current infection status. This limitation may partly explain differences between this study and investigations using stool antigen or urea breath tests, which detect active infection (10).

### Strengths and implications

A key strength of this study is its focus on an understudied population—Somali university students—for whom epidemiological data on *H. pylori* are limited. The findings provide baseline evidence on exposure patterns that may inform future research. However, public health implications should be regarded as exploratory. While the identified associations suggest potential areas for health education, causal inferences cannot be drawn, and the effectiveness of specific interventions cannot be assumed based on the present data.

### Summary

In summary, this study demonstrates a high seroprevalence of *H. pylori* among university students in Mogadishu and reports associations with selected lifestyle and environmental factors. These findings are descriptive and hypothesis-generating. Longitudinal studies incorporating multivariable adjustment and diagnostic methods capable of distinguishing active infection from past exposure are needed to clarify causal pathways and inform evidence-based strategies for *H. pylori* control among young adults in Somalia.

### Limitation

This study has several important limitations. First, ELISA IgG testing detects previous exposure rather than active infection, which may have resulted in misclassification and overestimation of prevalence. Second, lifestyle and behavioral variables were self-reported, introducing potential recall and social desirability bias. Third, the cross-sectional design precludes causal inference, limiting interpretation to associations only. Fourth, participants were recruited from a single university, increasing the likelihood of selection bias and limiting generalizability. Finally, regression analyses were unadjusted, and residual confounding cannot be excluded.

## Recommendations

Future studies should include multiple universities and larger samples to improve generalizability. Advanced diagnostic techniques such as stool antigen tests or urea breath tests should be used in combination with serology to better differentiate active from past seropositivity. Longitudinal or interventional studies are needed to explore causal pathways between lifestyle behaviors and *H. pylori* seropositivity. Public health efforts should focus on improving hygiene practices, promoting safe water use, encouraging regular sleep patterns, and raising awareness about modifiable dietary and lifestyle risks.

## Conclusion

This study demonstrates a high seroprevalence of *Helicobacter pylori* among students at Abrar University in Mogadishu, Somalia, indicating widespread prior exposure within this university population. The observed associations between *H. pylori* seropositivity and factors such as irregular sleep patterns, frequent consumption of spicy foods, and reliance on shared drinking water sources highlight the potential role of modifiable lifestyle and environmental exposures. However, these findings should be interpreted with caution, as the cross-sectional design precludes causal inference and the use of IgG serology reflects past exposure rather than active infection.

The results underscore the importance of targeted health education initiatives within university settings, focusing on hygiene practices, safe water use, and healthy lifestyle behaviors. This study provides baseline epidemiological evidence that may inform future multicenter, longitudinal research using diagnostic methods capable of distinguishing active seropositivity from previous exposure, thereby supporting more precise public health strategies for *H. pylori* control among young adults in Somalia.

## Data Availability

The original contributions presented in the study are included in the article/supplementary material, further inquiries can be directed to the corresponding author.

## References

[1] Hooi JKY Lai WY Ng WK Suen MMY Underwood FE Tanyingoh D . Global prevalence of *Helicobacter pylori* infection: systematic review and meta-analysis. Gastroenterology. (2021) 160:2181–2183.e1. doi: 10.1053/j.gastro.2021.02.014, 28456631

[2] Ndip RN MacKay WG Farthing MJG Weaver LT. Prevalence of *Helicobacter pylori* infection in asymptomatic individuals in Cameroon. J Clin Microbiol. (2004) 42:2639–42. doi: 10.1128/JCM.42.6.2639-2642.2004

[3] Alebie G Kaba D Yohannes B. Prevalence of *Helicobacter pylori* seropositivity and associated factors among university students in eastern Ethiopia. J Bacteriol Mycol Open Access. (2017) 5:00139. doi: 10.15406/jbmoa.2017.05.00139

[4] Enitan SS Ochei KC Oyekale OT. Screening for *Helicobacter pylori* among undergraduate students using serum antibody and stool antigen detection methods. Babcock Univ Med J. (2018) 1:1–7.

[5] Mnichil Z Nibret E Hailegebriel T Demelash M Mekonnen D. Prevalence and associated risk factors of *Helicobacter pylori* seropositivity in East Africa: a systematic review and meta-analysis. Braz J Microbiol. (2024) 55:51–64. doi: 10.1007/s42770-022-00866-1, 38040991 PMC10920553

[6] Khoder G Mina S Mahmoud I Muhammad JS Harati R Burucoa C. *Helicobacter pylori* seroprevalence in Tripoli, North Lebanon: assessment and risk factors. Biology (Basel). (2021) 10:599. doi: 10.3390/biology10070599, 34203570 PMC8301113

[7] Khoder G Muhammad JS Mahmoud I Soliman SSM Burucoa C. Prevalence of *helicobacter pylori* and its associated factors among healthy asymptomatic residents in the United Arab Emirates. Pathogens. (2019) 8:44. doi: 10.3390/pathogens8020044, 30939800 PMC6632043

[8] Öztekin C . Association of dietary habits with *Helicobacter pylori* seropositivity: evidence from adult populations. Diseases. (2021) 9:82. doi: 10.3390/diseases9040082, 34842655 PMC8628812

[9] Khoder G . Prevalence of *helicobacter pylori* and dietary risk factors in healthy adults in the middle east. Pathogens. (2021) 10:599. doi: 10.3390/pathogens10050599, 34068976 PMC8157079

[10] Smith SM O’Morain C McNamara D. Serological testing for *Helicobacter pylori*: advantages and limitations. World J Gastroenterol. (2020) 26:2819–32. doi: 10.3748/wjg.v26.i21.2819

